# Supplementary figures and images for: Cryo-EM structures of the caspase-activated protein XKR9 involved in apoptotic lipid scrambling
Source: eLife. 2021 Jul 15;10:e69800. doi: 10.7554/eLife.69800 (PMC8298096; doi:10.7554/eLife.69800)

Figure 1 – source data 1

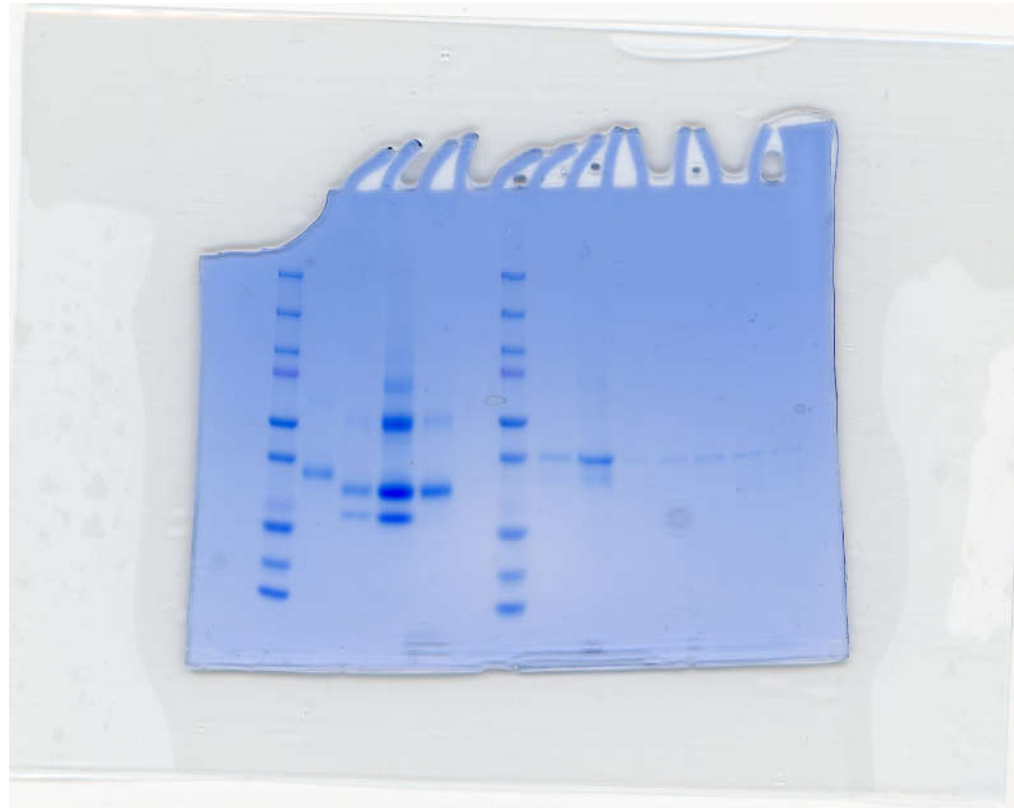

Gel lane shown in Figure 1A

\* full-length rXKR9

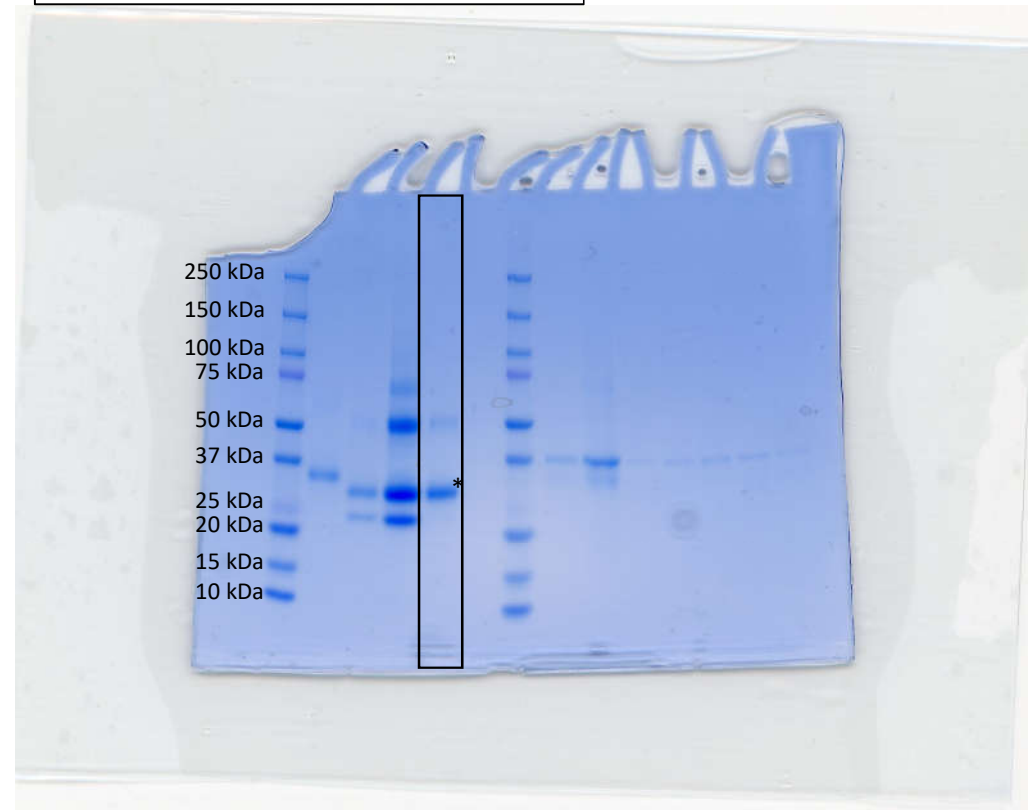

Supplement: Figure 1—source data 1. [file elife-69800-fig1-data1.zip › Figure_1_source_data_1.pdf]

Figure 1 – source data 2

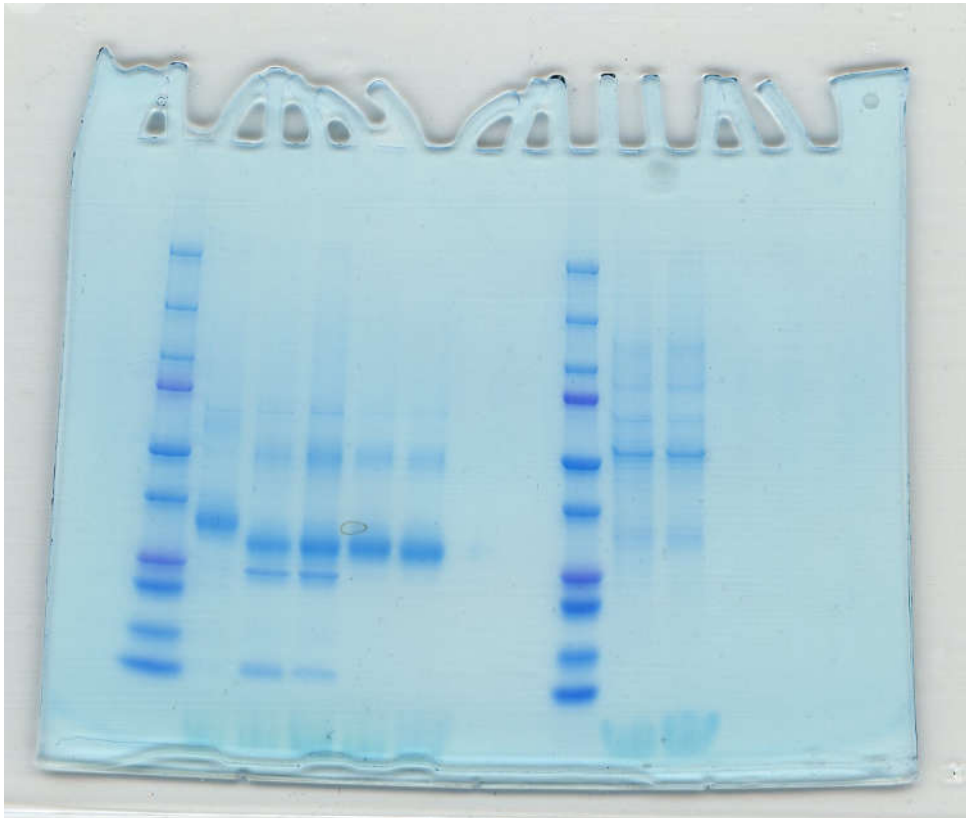

Gel lane shown in Figure 1B

\* caspase-3 treated rXKR9

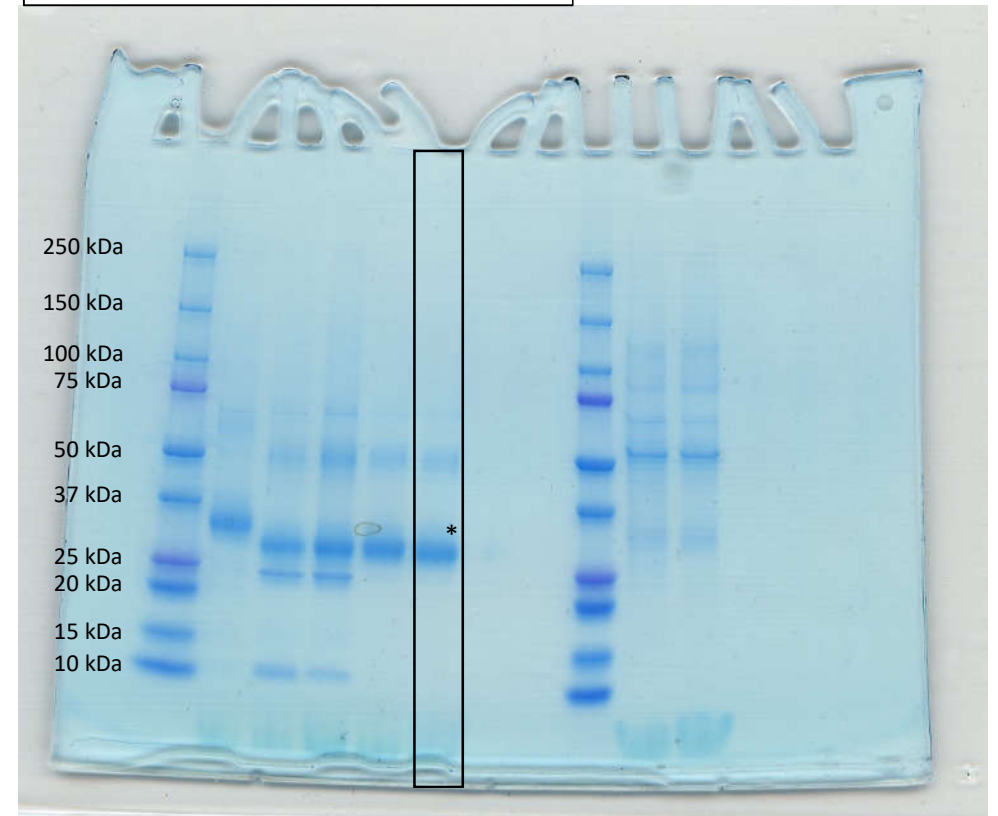

Supplement: Figure 1—source data 2. [file elife-69800-fig1-data2.zip › Figure_1_source_data_2.pdf]

Figure 1 – source data 3

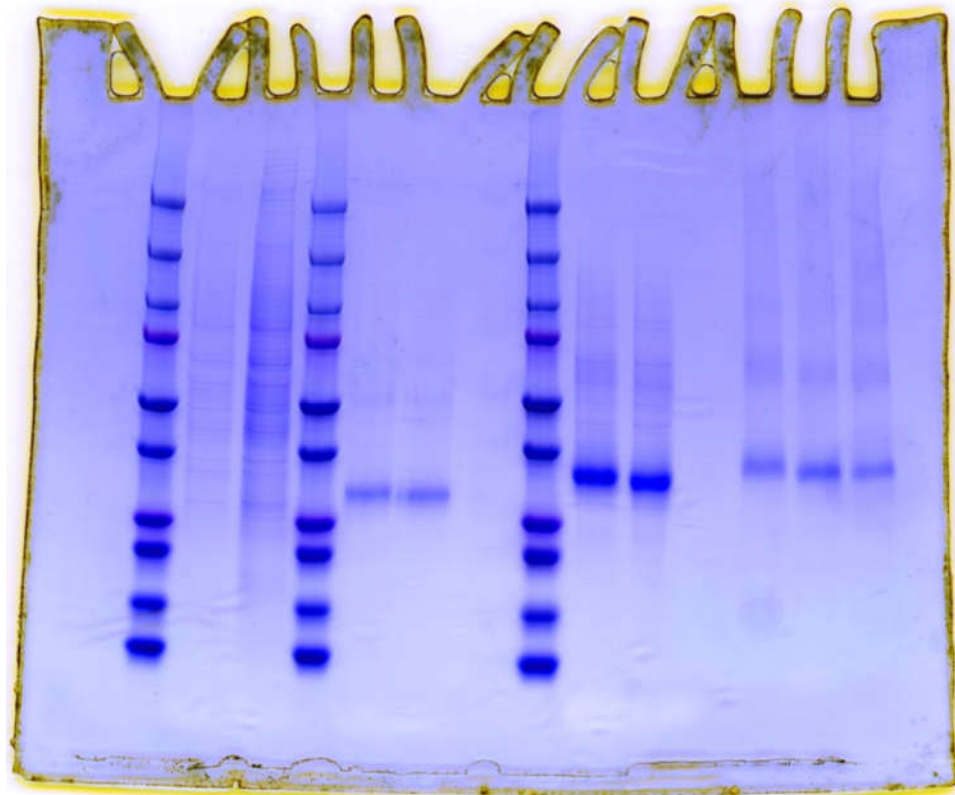

Gel lanes shown in Figure 1C

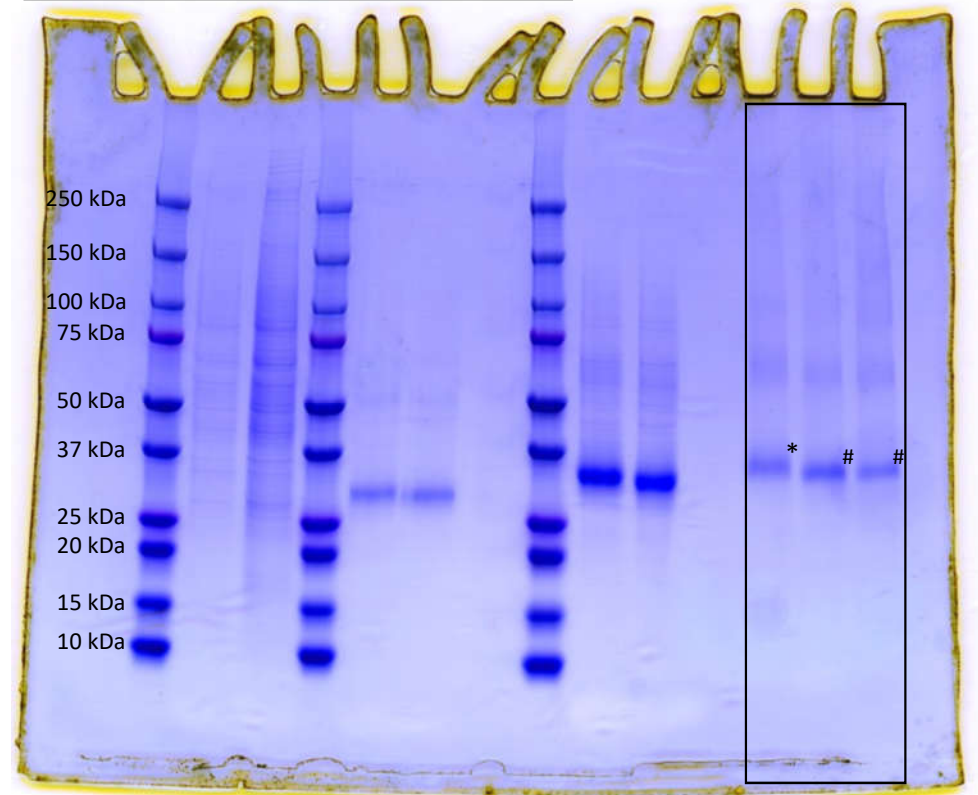

Supplement: Figure 1—source data 3. [file elife-69800-fig1-data3.zip › Figure_1_source_data_3.pdf]

Figure 1 – source data 5

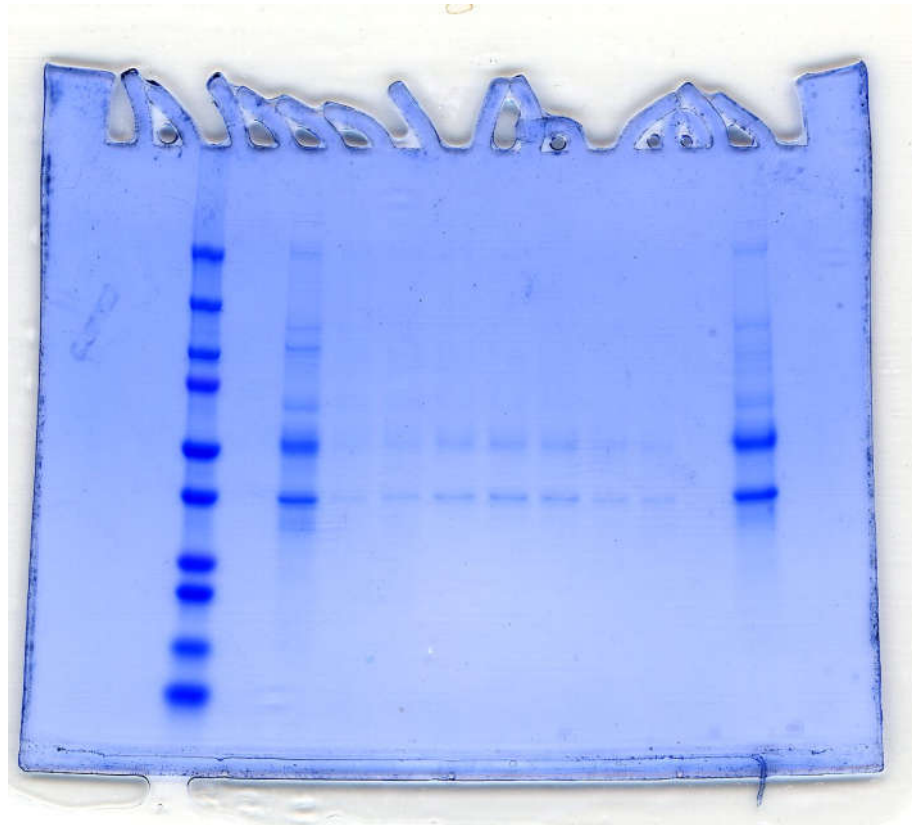

Gel lane shown in Figure 1E

\* XKR8  
# basigin

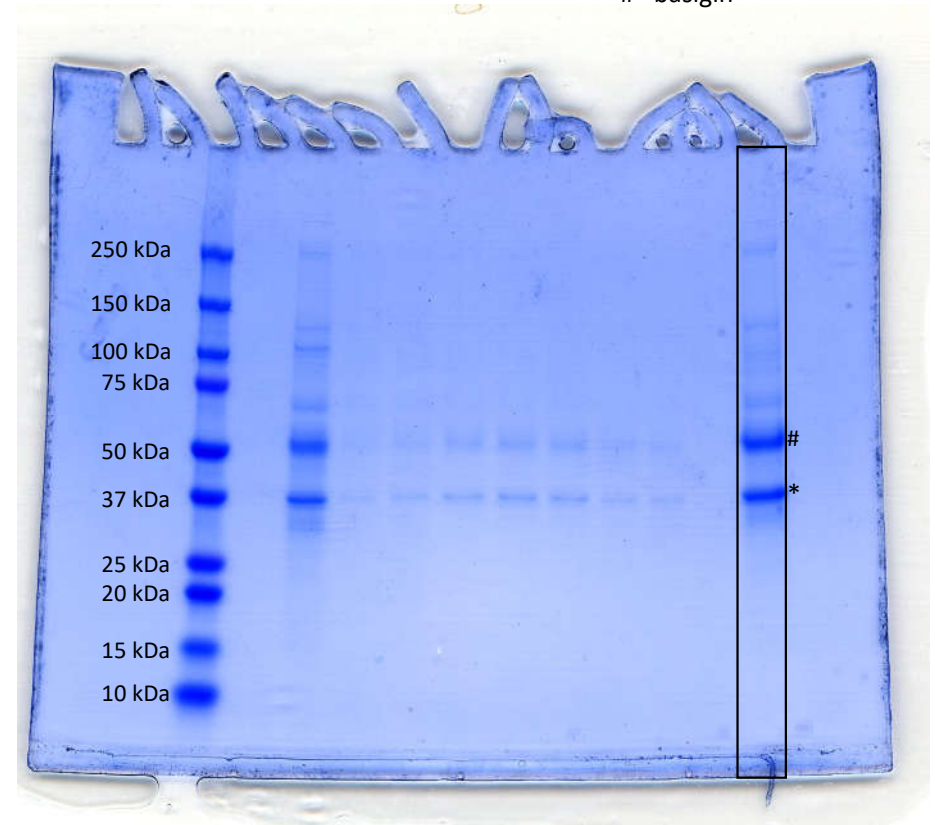

Supplement: Figure 1—source data 5. [file elife-69800-fig1-data5.zip › Figure_1_source_data_5.pdf]

Figure 1 – source data 6

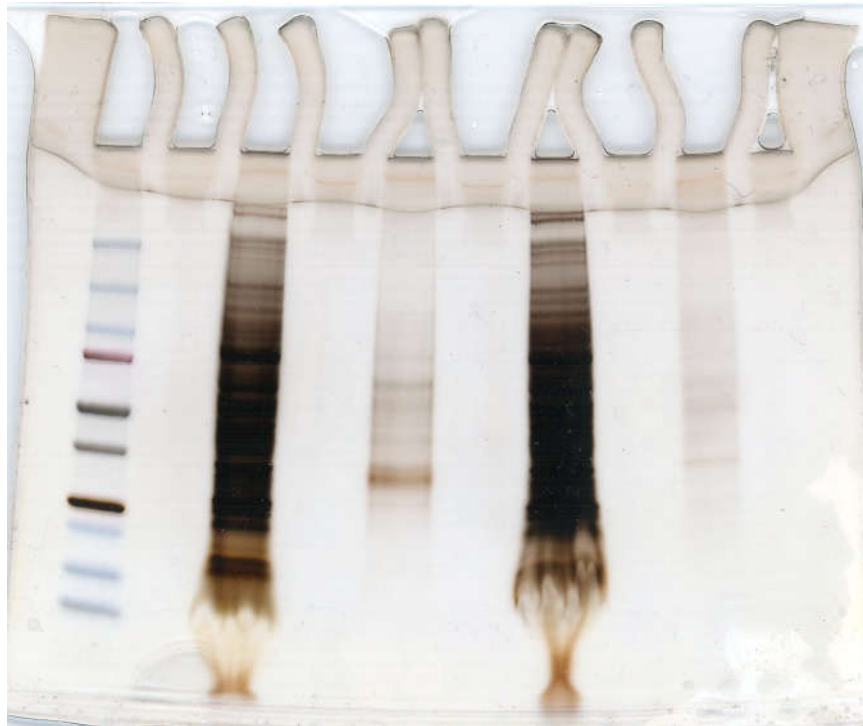

Gel lane shown in Figure 1G

\* rXKR9

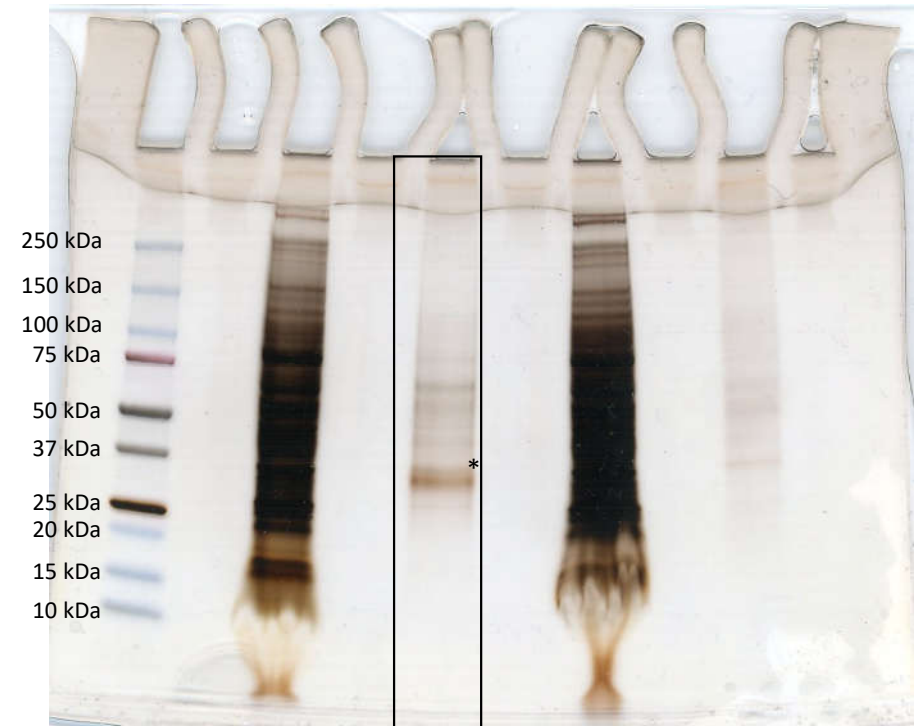

Supplement: Figure 1—source data 6. [file elife-69800-fig1-data6.zip › Figure_1_source_data_6.pdf]

Figure 1 – source data 7

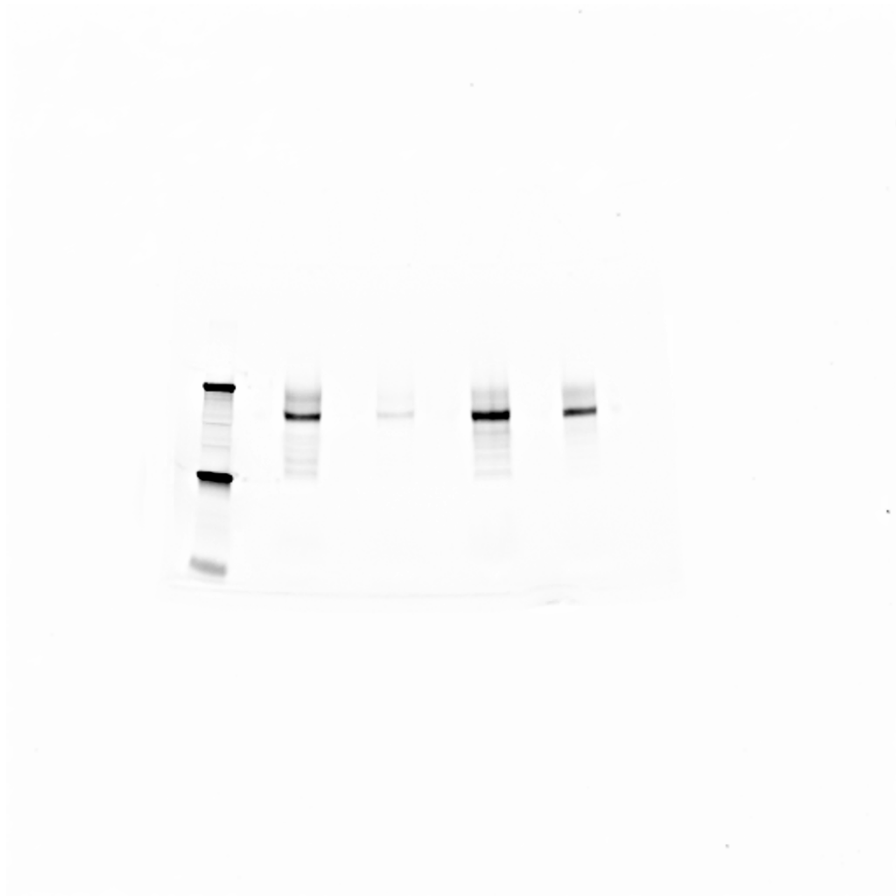

Gel lanes shown in Figure 1G

\* basigin

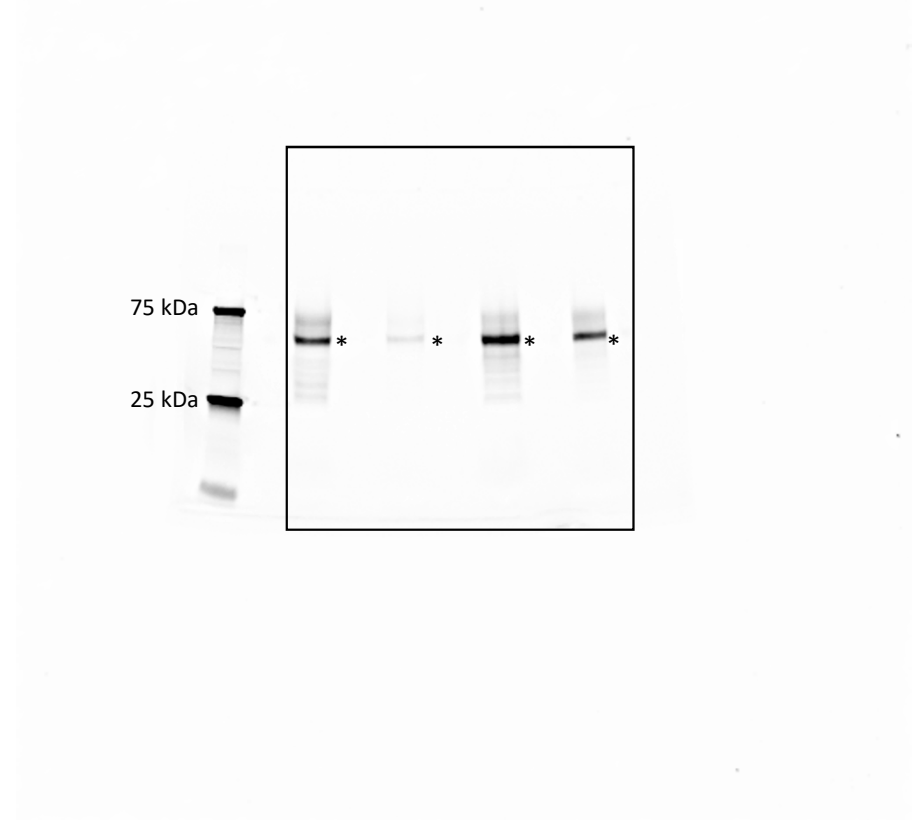

Supplement: Figure 1—source data 7. [file elife-69800-fig1-data7.zip › Figure_1_source_data_7.pdf]

Figure 1 – figure supplement 3 – source data 2

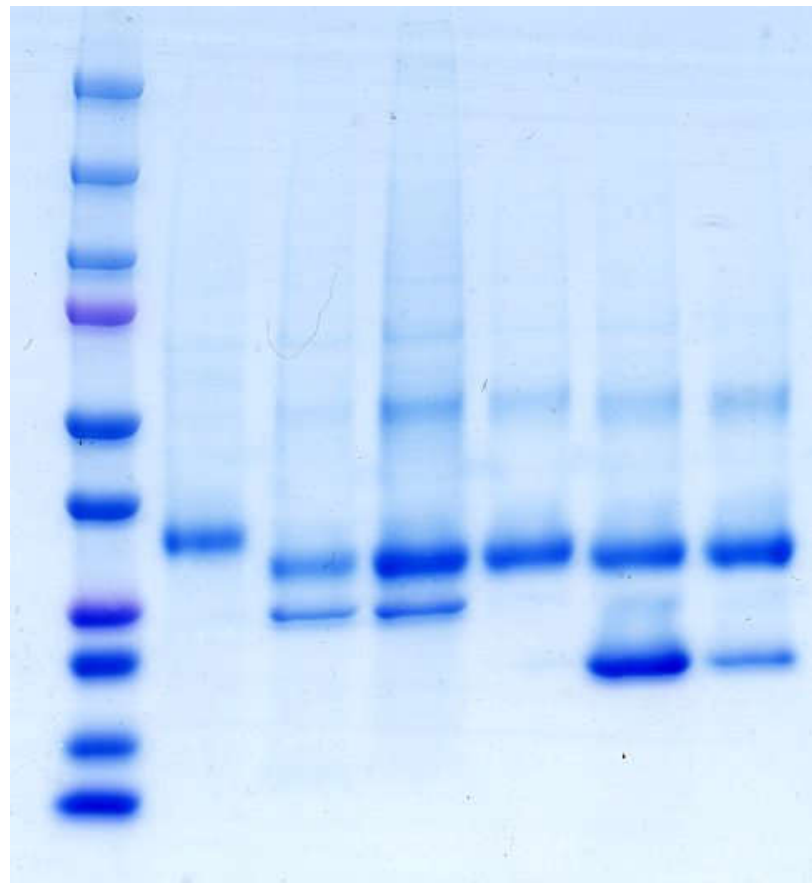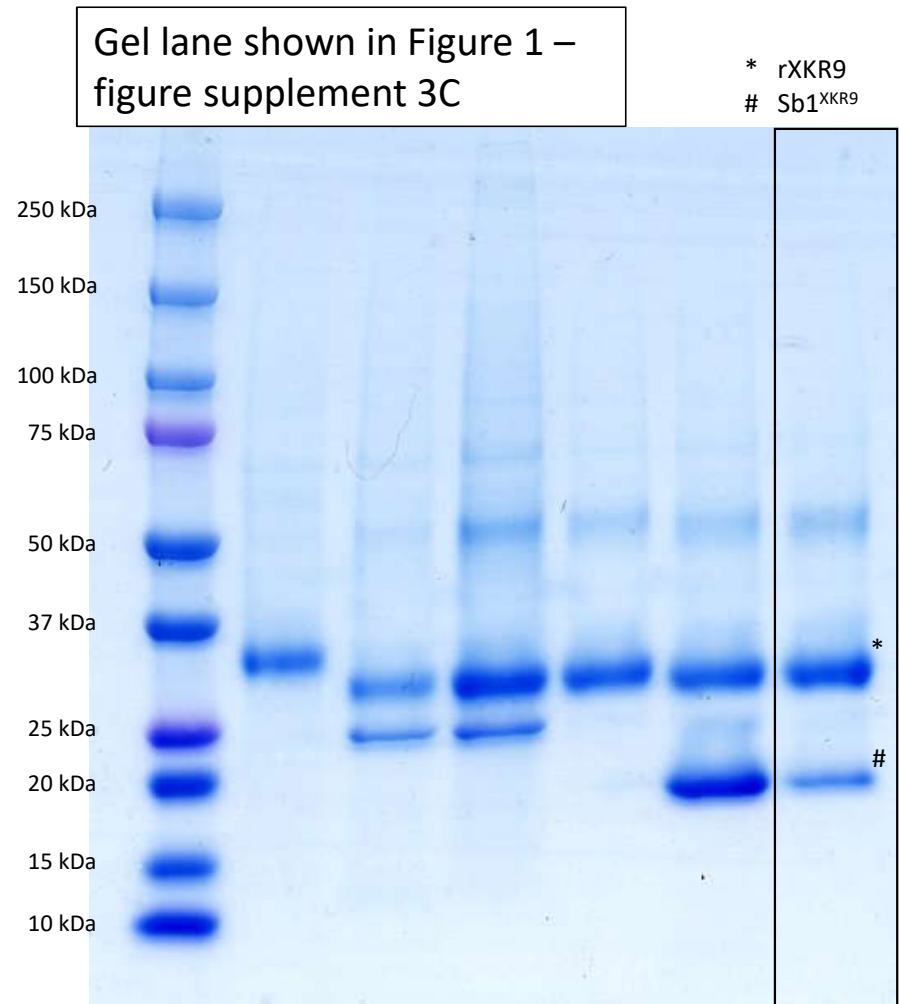

Supplement: Figure 1—figure supplement 2—source data 2. [file elife-69800-fig1-figsupp2-data2.zip › Figure_1_figure_supplement_3_source_data_2.pdf]
