# Supplementary material for: Cryo-EM structures of the caspase-activated protein XKR9 involved in apoptotic lipid scrambling
Source: eLife. 2021 Jul 15;10:e69800. doi: 10.7554/eLife.69800 (PMC8298096; doi:10.7554/eLife.69800)

Figure 1 – figure supplement 3 – source data 1

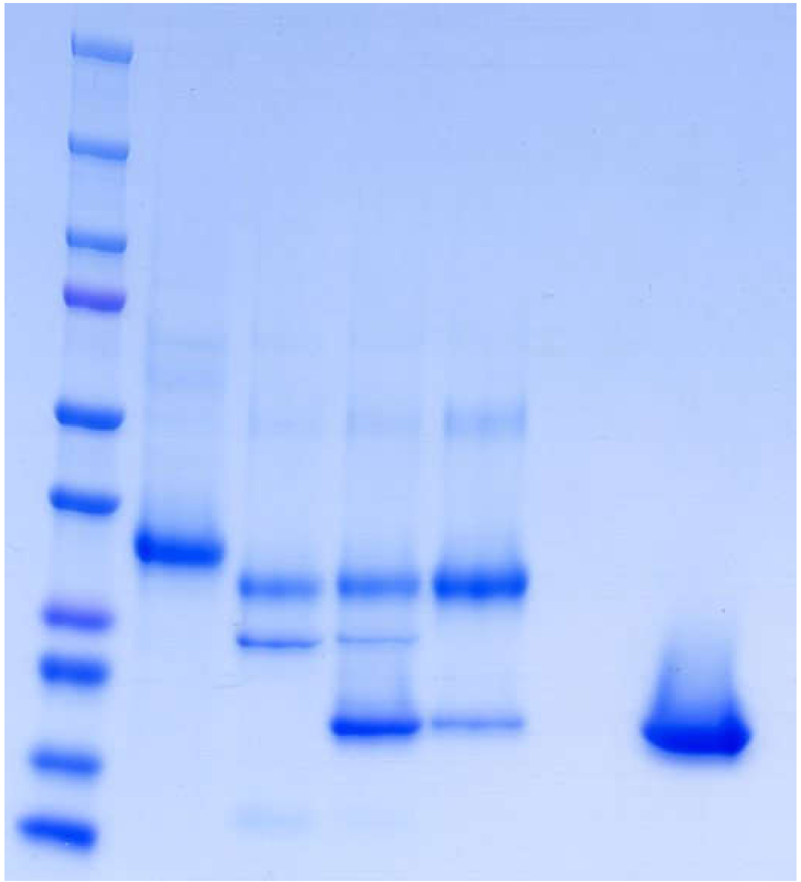

Gel lane shown in Figure 1 – figure supplement 3B

* rXKR9
\# Sb1^{XKR9}

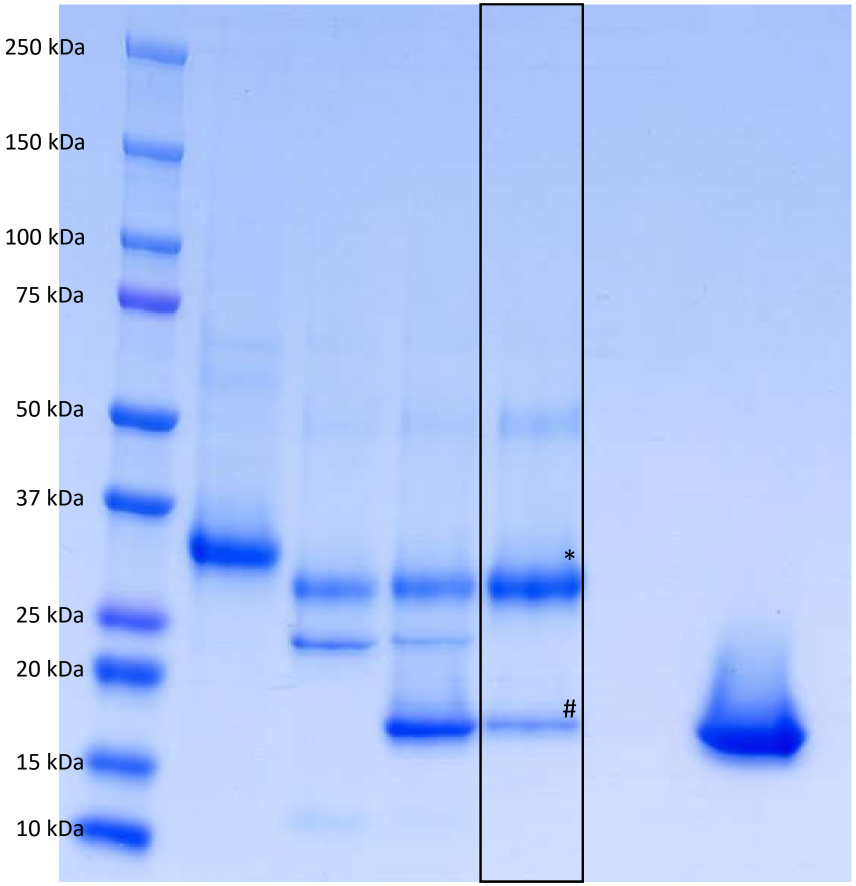

Supplement: Figure 1—figure supplement 2—source data 1. [file elife-69800-fig1-figsupp2-data1.zip › Figure_1_figure_supplement_3_source_data_1.pdf]
