# Supplementary material for: Cryo-EM structures of the caspase-activated protein XKR9 involved in apoptotic lipid scrambling
Source: eLife. 2021 Jul 15;10:e69800. doi: 10.7554/eLife.69800 (PMC8298096; doi:10.7554/eLife.69800)
Supplement: Transparent reporting form [file elife-69800-transrepform.docx]

***eLife’s* transparent reporting form**

We encourage authors to provide detailed information *within their submission* to facilitate the interpretation and replication of experiments. Authors can upload supporting documentation to indicate the use of appropriate reporting guidelines for health-related research (see [EQUATOR Network](http://www.equator-network.org/%20)), life science research (see the [BioSharing Information Resource](https://biosharing.org/)), or the [ARRIVE guidelines](http://www.plosbiology.org/article/info:doi/10.1371/journal.pbio.1000412) for reporting work involving animal research. Where applicable, authors should refer to any relevant reporting standards documents in this form.

If you have any questions, please consult our Journal Policies and/or contact us: [editorial@elifesciences.org](mailto:editorial@elifesciences.org).

**Sample-size estimation**

- You should state whether an appropriate sample size was computed when the study was being designed
- You should state the statistical method of sample size computation and any required assumptions
- If no explicit power analysis was used, you should describe how you decided what sample (replicate) size (number) to use

Please outline where this information can be found within the submission (e.g., sections or figure legends), or explain why this information doesn’t apply to your submission:

- For the scrambling and proteoliposome data in Figure 1D and S1C, data from 3 technical replicates, including errors, are shown for a single representative proteoliposome reconstitution batch. Several reconstitution trials using separate batches of purified protein and synthetic lipid lots resulted in similar data.
- The sample size for cryo-EM analysis was chosen to obtain the best possible resolution for each dataset. The detailed procedure is outlined in materials and methods section on cryo-EM data collection and image processing

**Replicates**

- You should report how often each experiment was performed
- You should include a definition of biological versus technical replication
- The data obtained should be provided and sufficient information should be provided to indicate the number of independent biological and/or technical replicates
- If you encountered any outliers, you should describe how these were handled
- Criteria for exclusion/inclusion of data should be clearly stated
- High-throughput sequence data should be uploaded before submission, with a private link for reviewers provided (these are available from both GEO and ArrayExpress)

Please outline where this information can be found within the submission (e.g., sections or figure legends), or explain why this information doesn’t apply to your submission:

- Purifications and co-purifications were performed at least two times and resulted in similar data
- For proteo-liposome experiments, the three technical replicates refer to three separate samples prepared and analyzed separately from a single batch of reconstituted proteo-liposomes
- No obvious outliers were encountered or omitted from presented data
- Presented data is in accordance with eLife’s regulations and appropriate information regarding the number and type of replicates is provided in figure legends

**Statistical reporting**

- Statistical analysis methods should be described and justified
- Raw data should be presented in figures whenever informative to do so (typically when N per group is less than 10)
- For each experiment, you should identify the statistical tests used, exact values of N, definitions of center, methods of multiple test correction, and dispersion and precision measures (e.g., mean, median, SD, SEM, confidence intervals; and, for the major substantive results, a measure of effect size (e.g., Pearson's r, Cohen's d)
- Report exact p-values wherever possible alongside the summary statistics and 95% confidence intervals. These should be reported for all key questions and not only when the p-value is less than 0.05.

Please outline where this information can be found within the submission (e.g., sections or figure legends), or explain why this information doesn’t apply to your submission:

- For scrambling assays, average and SEM of three technical replicates were calculated, as described in the figure legends of Figure 1D and S1C
- For cryo-EM maps, the resolution was estimated the gold-standard Fourier shell correlation between two independently refined half-maps. For details, see material and method section

(For large datasets, or papers with a very large number of statistical tests, you may upload a single table file with tests, Ns, etc., with reference to sections in the manuscript.)

**Group allocation**

- Indicate how samples were allocated into experimental groups (in the case of clinical studies, please specify allocation to treatment method); if randomization was used, please also state if restricted randomization was applied
- Indicate if masking was used during group allocation, data collection and/or data analysis

Please outline where this information can be found within the submission (e.g., sections or figure legends), or explain why this information doesn’t apply to your submission:

NA

**Additional data files (“source data”)**

- We encourage you to upload relevant additional data files, such as numerical data that are represented as a graph in a figure, or as a summary table
- Where provided, these should be in the most useful format, and they can be uploaded as “Source data” files linked to a main figure or table
- Include model definition files including the full list of parameters used
- Include code used for data analysis (e.g., R, MatLab)
- Avoid stating that data files are “available upon request”

Please indicate the figures or tables for which source data files have been provided:

Source data has been provided for following figures:
Figure 1A-E and G, Figure 1-figure supplement 1C, Figure 1-figure supplement 3B-D.
